# Suboptimal Effectiveness of the 2011–2012 Seasonal Influenza Vaccine in Adult Korean Populations

**DOI:** 10.1371/journal.pone.0098716

**Published:** 2015-03-27

**Authors:** Won Suk Choi, Ji Yun Noh, Ji Hyeon Baek, Yu Bin Seo, Jacob Lee, Joon Young Song, Dae Won Park, Jin Soo Lee, Hee Jin Cheong, Woo Joo Kim

**Affiliations:** 1 Division of Infectious Diseases, Department of Internal Medicine, Korea University College of Medicine, Seoul, Republic of Korea; 2 Division of Infectious Diseases, Department of Internal Medicine, Inha University College of Medicine, Icheon, Republic of Korea; 3 Division of Infectious Diseases, Department of Internal Medicine, Hallym University College of Medicine, Chuncheon, Republic of Korea; 4 Transgovernmental Enterprise for Pandemic Influenza in Korea, Seoul, Republic of Korea; Centers for Disease Control and Prevention, UNITED STATES

## Abstract

**Background:**

The effectiveness of the 2011–2012 seasonal influenza vaccine was evaluated in adult Korean populations with regard to how well it could prevent laboratory-confirmed influenza and influenza-related complications.

**Materials and Methods:**

A retrospective case-control and retrospective cohort study was conducted among patients who visited four selected hospitals from September 2011 to May 2012. The analysis included 1,130 laboratory-confirmed influenza patients. For each influenza case, one control patient was chosen at a ratio of 1:1. A control was defined as an age group-matched patient who visited the same hospital with influenza-like illness within 48 hours of symptom onset but for whom laboratory tests were negative for influenza. Age group and visit date were matched between the cases and controls. Vaccine effectiveness (VE) was defined as [100 × (1-odds ratio for influenza in vaccinated versus non-vaccinated persons)]. The patients with laboratory-confirmed influenza were followed for at least one month through reviewing the medical records and conducting a telephone interview.

**Results:**

The VE of the 2011–2012 seasonal influenza vaccine was 3.8% [95% confidence interval (CI), -16.5% to 20.6%] for preventing laboratory-confirmed influenza, -16.1% (95% CI, -48.3 to 9.1) for influenza A and 26.2% (95% CI, -2.6 to 46.2) for influenza B. The age-specific adjusted VE was 0.3% (95% CI, -29.4 to 23.1) among participants aged 19 to 49 years, 11.9% (95% CI, -34.3 to 42.2) among those aged 50 to 64 years and -3.9% (-60.1 to 32.5) among those aged ≥65 years. The adjusted VE for preventing any influenza-related complications was -10.7% (95% CI, -41.1% to 42.2%).

**Conclusions:**

The 2011–2012 seasonal influenza vaccine was not effective in preventing laboratory-confirmed influenza or influenza-related complications in adult Korean populations.

## Introduction

The seasonal influenza vaccine has been distributed very actively in Korea. According to a report of the Macroepidemiology of Influenza Vaccination Study Group, Korea has the second largest supply of influenza vaccine of any country [1]. In particular, influenza vaccination coverage is high in the elderly due to government reimbursement [2]. During the 2011–2012 season, more than 20 million doses of influenza vaccines were approved for use by the Korea Food and Drug Administration. Among those vaccines, 96.3% was the traditional unadjuvanted trivalent vaccine. Other types of influenza vaccines accounted for only 3.7%; 1.0% was live attenuated vaccine, 1.4% was adjuvanted vaccine and 1.3% was inactivated vaccine to be administered through an intradermal route [3].

Although the annual influenza vaccination coverage rate is not monitored regularly, the need for the influenza vaccine seems to be increasing since the 2009 influenza pandemic. However, previous studies on the effectiveness of the influenza vaccine reported conflicting results, especially in the elderly [4–6]. Considering the large supply of influenza vaccine in Korea, a regular and accurate assessment is needed. Because of the large demand, annual assessment of the effectiveness of the seasonal influenza vaccine began during the 2010–2011 influenza season with the support of the Transgovernmental Enterprise for Pandemic Influenza in Korea (TEPIK). Herein, we evaluate the effectiveness of the 2010–2011 seasonal influenza vaccine in a population of Korean adults.

## Materials and Methods

This study was performed sequentially following the 2010–2011 season with the same methods as described in previous reports [7–9].

### Study population and data collection

A retrospective case-control study was conducted among patients who visited four university hospitals with influenza-like illnesses (ILI) from September 2011 to May 2012. ILI was defined as fever with cough, sore throat or rhinorrhea. The patients with ILI were usually tested for influenza at the participating hospitals according to the physician’s decision, typically in the emergency or outpatient department. If a patient was inpatient and ILI occurred after more than 48 hours of hospitalization, the patient was excluded from our study. Patients under 18 years of age were also excluded. Laboratory-confirmed influenza was defined as a positive result from a rapid antigen test (RAT), polymerase chain reaction (PCR) or influenza virus culture, regardless of when the symptoms started. For each patient with laboratory-confirmed influenza, one control patient was chosen at a ratio of 1:1. A control was defined as an age group-matched patient who visited the same hospital with ILI within 48 hours of symptom onset but for whom laboratory tests were negative for influenza. Age group and visit dates were matched between cases and controls. All participants were stratified into three age groups: 18 to 50, 51 to 64 and over 65 years. If two or more control participants fulfilled the matching criteria, the one with the smallest age difference was selected. If a selected control participant had an obscure influenza vaccination history or refused to take part in a telephone interview, a new control was chosen using the same criteria.

For the patients with laboratory-confirmed influenza, retrospective cohort study was performed to assess the vaccine effectiveness (VE) for preventing influenza-related complications. The patients were followed for one month through reviewing the medical records and conducting a telephone interview.

### Data collection

Using a standardized questionnaire, the following data were collected for the participants by reviewing their medical records: age, sex, clinical symptoms, date of symptom onset, date of clinic visits, vaccination status for the 2011–2012 season, diagnostic laboratory results for influenza, chronic medical conditions, pregnancy and smoking status (current, previous or non-smoker). The patients were defined as having a chronic medical condition if they had any of the following: diabetes mellitus, cardiovascular disease, cerebrovascular disease, neuromuscular disease, chronic pulmonary disease, chronic renal disease, chronic hepatic disease, current treatment for malignancy, congenital or acquired immunodeficiency, and medication with immunosuppressant agents. The 2011–2012 seasonal influenza vaccination history for each participant was checked by reviewing the medical records and conducting a telephone interview. We defined vaccinated individuals as those who had received the seasonal influenza vaccine at least 14 days or more from the date of symptom onset. To assess the prognosis of influenza according to the 2011–2012 seasonal influenza vaccination history, the following data were collected during the follow-up period: antiviral use, hospitalization in a general ward or intensive care unit, duration of hospitalization, occurrence of influenza-related complications, including exacerbation of underlying diseases, and death within 30 days from the diagnosis of influenza.

### Laboratory analysis

All four hospitals followed the same laboratory methods for confirmation of influenza. Nasopharyngeal or throat swab specimens were used for the influenza laboratory tests. RAT was performed using a commercial kit, SD Bioline Influenza Antigen Test (Standard Diagnostic, Inc., Korea), according to the manufacturer’s instructions. PCR was performed using a commercial multiplex real-time PCR kit, Anyplex II RV16 Detection (Seegene, Korea). An influenza virus culture was carried out using an R-Mix Too (A549/MDCK) shell vial culture. After the virus culture, immunofluorescence staining was performed using a Respiratory Virus Screening and ID Kit (Dow Biomedical, Korea) to identify the virus.

### Data analyses

Statistical analyses were performed using the Statistical Package for the Social Sciences (SPSS) version 20.0 (SPSS, Chicago, IL, US). Logistic regression was used to estimate the odds ratio for laboratory-confirmed influenza or influenza-related complications in vaccinated versus unvaccinated participants. The VE was defined as [100 × (1-odds ratio for influenza in vaccinated versus non-vaccinated persons)]. Logistic regression models were adjusted for age, comorbidities and hospitals in determining the VE for preventing laboratory-confirmed influenza. The models were adjusted for age, comorbidities, antiviral use and hospitals in determining the VE for preventing influenza-related complications. The age was adjusted as a continuous variable. Each comorbidity was adjusted as individual variable. The variables used for the adjustment for the model were chosen in the initial stage of the study design with the assumption that those would affect the results. The significant difference in the distribution of variables between cases and controls was estimated by the Chi-squared or the Fisher’s exact test for categorical variables and Student’s t-test for quantitative variables. A bilateral p<0.05 value was considered a significant result.

### Review of the research plan

The study was performed with approval of the Institution Review Board from each of the four hospitals: Korea University Guro Hospital Institutional Review Board, Korea University Ansan Hospital Institutional Review Board, Inha University Hospital Institutional Review Board and Kangnam Sacred Heart Hospital Institutional Review Board/Ethics Committee. Written informed consent was waived because most data were collected retrospectively by reviewing medical records and a telephone interview was done only to confirm the 2011–2012 seasonal influenza vaccination history. Given the characteristics of the study design, it was not practical and technically difficult to obtain written informed consent. However, the details of the study including study objective, process, data handling and etc. were explained to the study participants during the telephone interview. If a participant refused to have a telephone interview or to be included in the study, he or she was excluded from the study. The participant consents were recorded as a document. The Institution Review Boards of the four hospitals approved the procedure.

## Results

### Characteristics of study participants

During the 2011–2012 influenza season, 1,570 patients were diagnosed with laboratory-confirmed influenza in four selected hospitals. Among them, 440 patients were excluded due to an obscure influenza vaccination history or a hospital-acquired infection. As a result, 1,130 laboratory-confirmed influenza patients were included in the study. Among the selected participants, 1,125 (99.6%) were tested with RAT and, of those patients, 1,035 (92.0%) were positive for influenza; 206 (18.2%) were tested with PCR and, of those participants, 174 (71.4%) were positive for influenza; and 51 (4.5%) were tested with virus culture and, of those participants, 33 (64.7%) were positive for influenza. All selected patients were successfully followed for one month.

During the influenza season, 7,390 patients had one or more laboratory tests for influenza that were negative. Among them, 1,130 patients were selected as the controls according to the matching criteria; 1,118 (98.9%) of the selected control participants were tested with RAT, while 122 (10.8%) were tested with PCR, and 29 (2.6%) were tested with virus culture.

Among the case participants, 678 (60.0%) were positive for influenza A, 435 (38.5%) were positive for influenza B and 17 (1.5%) were positive for both influenza A and B (Table 1). Six hundred and eighty five (60.6%) cases were younger than 50 years of age, 224 (19.8%) cases were 50 to 64 years of age and 221 (19.6%) cases were ≥65 years of age. Five hundred and fifty four (40.2%) cases were male, and 467 (41.3%) cases had one or more underlying disease. There was no significant difference in gender, smoking history or frequency of chronic medical conditions between the experimental and control groups. However, pregnancy was more common in the case group. Solid organ cancer and the use of immunosuppressive agents were more prevalent in the control group.

**Table 1:** Demographic characteristics of the study participants.

	Patients with laboratory-confirmed influenza, N = 1,130 (%)	Patients with negative laboratory tests, N = 1,130 (%)	*p*-value
Gender, male	454 (40.2)	453 (40.1)	1.00
Influenza subtype			
A	678 (60.6)	-	-
B	435 (38.5)	-	-
Both A & B ^a^	17 (1.5)	-	-
Age groups			1.00
19 to 49 years	685 (60.6)	685 (60.6)	
50 to 64 years	224 (19.8)	224 (19.8)	
≥ 65 years	221 (19.6)	221 (19.6)	
Smoker status			0.14
Current smoker	136 (12.0)	156 (13.8)	
Ex-smoker	96 (8.5)	76 (6.7)	
Non-smoker	701 (62.0)	675 (59.7)	
Comorbidities ^b^	467 (41.3)	494 (43.7)	0.27
Diabetes mellitus	121 (10.7)	102 (9.0)	0.20
Hypertension	222 (19.6)	225 (19.9)	0.92
Cardiovascular diseases	76 (6.7)	74 (6.5)	0.93
Cerebrovascular diseases	36 (3.2)	27 (2.4)	0.31
Neuromuscular diseases	5 (0.4)	1 (0.1)	0.22
Chronic pulmonary diseases	75 (6.6)	72 (6.4)	0.87
Chronic renal failure	33 (2.9)	23 (2.0)	0.22
Chronic liver diseases	40 (3.5)	39 (3.5)	1.00
Solid organ cancer	56 (5.0)	81 (7.2)	0.03
Hematologic malignancy	7 (0.6)	11 (1.0)	0.48
Taking immunosuppressants	15 (1.3)	35 (3.1)	<0.01
Pregnancy	37 (3.3)	20 (1.8)	0.03
Long-term care facility residence	11 (1.0)	5 (0.4)	0.21
2011–2012 season influenza vaccination history	392 (34.7)	392 (34.7)	1.00
Vaccination history confirmed through medical records	212 (18.8)	202 (17.9)	0.65

^a^ Seventeen (1.5%) participants were positive for both influenza A and B in the rapid antigen test. The cases were suspected to be influenza A and B co-infections according to the manufacturer’s instructions.

^b^ Some patients had one or more comorbidities.

### The effectiveness at preventing laboratory-confirmed influenza

Among the 2,260 participants, 784 (34.7%) were vaccinated with a dose of 2011–2012 seasonal influenza vaccine. There was no difference in the influenza vaccination rate between the two groups. The adjusted VE of the 2011–2012 seasonal influenza vaccine was calculated as 3.8% (95% confidence interval [CI], -16.5% to 20.6%) for all influenza types, -16.1% (95% CI, -48.3% to 9.1%) for influenza A, and 26.2% (95% CI, -2.6% to 46.2%) for influenza B (Table 2). The age-specific adjusted VE was 0.3% (95% CI, -29.4% to 23.1%) among participants from 19 to 49 years of age, 11.9% (95% CI, -34.3% to 42.2%) among those aged 50 to 64 years and -3.9% (95% CI, -60.1% to 32.5%) among those aged 65 years or older.

**Table 2:** Vaccine effectiveness of the 2010–2011 influenza vaccine for preventing laboratory-confirmed influenza according to the influenza type and the age group.

		Case, N (%)	Control, N (%)	Unadjusted vaccine effectiveness, % (95% CI)	Adjusted vaccine effectiveness, % (95% CI)
Influenza A	19–49 years	374	374	-21.8 (-71.5 to 13.5)	-23.0 (-75.1 to 13.6)
	Vaccinees	92 (24.6)	79 (21.1)		
	Unvaccinees	282 (75.4)	295 (78.9)		
	50–64 years	141	141	-13.2 (-84.5 to 30.5)	-9.5 (-86.6 to 35.7)
	Vaccinees	52 (36.9)	48 (34.0)		
	Unvaccinees	89 (63.1)	93 (66.0)		
	≥ 65 years	163	163	-25.1 (-99.1 to 21.4)	-13.7 (-88.9 to 31.5)
	Vaccinees	114 (69.9)	106 (65.0)		
	Unvaccinees	49 (30.1)	57 (35.0)		
	Overall age	678	678	-17.3 (-46.4 to 6.0)	-16.1 (-48.3 to 9.1)
	Vaccinees	258 (38.1)	233 (34.4)		
	Unvaccinees	420 (61.9)	445 (65.6)		
Influenza B	19–49 years	301	301	22.0 (-14.5 to 46.9)	20.4 (-20.5 to 47.5)
	Vaccinees	61 (20.3)	74 (24.6)		
	Unvaccinees	240 (79.7)	227 (75.4)		
	50–64 years	79	79	39.2 (-17.2 to 68.4)	55.1 (-0.8 to 80.0)
	Vaccinees	24 (30.4)	33 (41.8)		
	Unvaccinees	55 (69.6)	46 (58.2)		
	≥ 65 years	55	55	28.2 (-81.1 to 71.5)	28.7 (-101.8 to 74.8)
	Vaccinees	42 (76.4)	45 (81.8)		
	Unvaccinees	13 (23.6)	10 (18.2)		
	Overall age	435	435	23.2 (-2.1 to 42.3)	26.2 (-2.6 to 46.2)
	Vaccinees	127 (29.2)	152 (34.9)		
	Unvaccinees	308 (70.8)	283 (65.1)		
Overall influenza	19–49 years	685	685	0 (-28.7 to 22.3)	0.3 (-29.4 to 23.1)
	Vaccinees	157 (22.9)	157 (22.9)		
	Unvaccinees	528 (77.1)	528 (77.1)		
	50–64 years	224	224	9.3 (-33.7 to 38.5)	11.9 (-34.3 to 42.2)
	Vaccinees	76 (33.9)	81 (36.2)		
	Unvaccinees	148 (66.1)	143 (63.8)		
	≥ 65 years	221	221	-11.6 (-68.2 to 36.0)	-3.9 (-60.1 to 32.5)
	Vaccinees	159 (71.9)	154 (69.7)		
	Unvaccinees	62 (28.1)	67 (30.3)		
	Overall age	1130	1130	0 (-18.9 to 15.9)	3.8 (-16.5 to 20.6)
	Vaccinees	392 (34.7)	392 (34.7)		
	Unvaccinees	738 (65.3)	738 (65.3)		

CI, confidence interval

### The effectiveness at preventing influenza-related complications

Among laboratory-confirmed influenza cases, 172 (15.2%) had one or more influenza-related complication including pneumonia, asthma exacerbation, chronic obstructive pulmonary disease exacerbation, myocardiac infarction, acute renal failure, encephalopathy, and rhabdomyolysis. The most common complication was pneumonia (124 cases, 72.1%). The incidence of the influenza-related complications according to age group is 5.4% among participants aged 19 to 49 years, 17.4% among those aged 50 to 64 years and 43.4% among those aged 65 years or more. Two hundred thirty-one cases (20.4%) were hospitalized due to influenza, and 25 (2.2%) cases were admitted to intensive care units. Four (0.4%) cases died within 30 days after hospitalization. The adjusted VE of the 2011–2012 seasonal influenza vaccine for preventing any influenza-related complication was -10.7% (95% CI, -41.1% to 42.2%) (Table 3). The 2011–2012 seasonal influenza vaccine did not demonstrate statistically significant effectiveness in preventing pneumonia, hospitalization or death. The subgroup analysis by influenza virus type also did not demonstrate a statistically significant VE.

**Table 3:** Vaccine effectiveness of the 2010–2011 influenza vaccine for preventing influenza-related complications and hospitalization.

		Case, N (%)	Control,N (%)	Unadjusted vaccine effectiveness, % (95% CI)	Adjusted vaccine effectiveness, % (95% CI)
Overall influenza-related complications ^a^	Influenza A	122	556	-114.5 (-218.8 to -44.3)	-15.9 (-100.6 to 33.0)
	Vaccinees	65 (53.3)	193 (34.7)		
	Unvaccinees	57 (46.7)	363 (65.3)		
	Influenza B	46	389	-235.0 (-524.3 to -79.7)	-29.2 (-207.8 to 45.8)
	Vaccinees	25 (54.3)	102 (26.2)		
	Unvaccinees	21 (45.7)	287 (73.8)		
	Overall influenza	172	958	-152.2 (-250.6 to -81.5)	-10.7 (-41.1 to 42.2)
	Vaccinees	92 (53.5)	300 (31.3)		
	Unvaccinees	80 (46.5)	658 (68.7)		
Pneumonia	Influenza A	87	591	-80.3 (-183.4 to -14.7)	-46.5 (-163.4 to 18.5)
	Vaccinees	44 (50.6)	214 (36.2)		
	Unvaccinees	43 (49.4)	377 (63.8)		
	Influenza B	34	401	-243.6 (-600.4 to -68.6)	-54.7 (-299.3 to 40.0)
	Vaccinees	19 (55.9)	108 (26.9)		
	Unvaccinees	15 (44.1)	293 (73.1)		
	Overall influenza	124	1006	-128.8 (-233.3 to -57.0)	-26.2 (-104.0 to 21.9)
	Vaccinees	65 (52.4)	327 (32.5)		
	Unvaccinees	59 (47.6)	679 (67.5)		
Hospitalization	Influenza A	166	512	-150.7 (-258.3 to -75.4)	-4.1 (-72.5 to 37.2)
	Vaccinees	91 (54.8)	167 (32.6)		
	Unvaccinees	75 (45.2)	345 (67.4)		
	Influenza B	61	374	-224.1 (-463.8 to -86.3)	-35.6 (-180.1 to 34.4)
	Vaccinees	32 (52.5)	95 (25.4)		
	Unvaccinees	29 (47.5)	279 (74.6)		
	Overall influenza	231	899	-185.6 (-283.9 to -112.4)	-16.4 (-73.2 to 21.7)
	Vaccinees	126 (54.5)	327 (32.5)		
	Unvaccinees	105 (45.5)	633 (70.4)		
Hospitalization to ICU	Influenza A	19	659	-187.8 (-640.8 to -11.8)	-11.6 (-268.7 to 66.2)
	Vaccinees	12 (63.2)	246 (37.3)		
	Unvaccinees	7 (36.8)	413 (62.7)		
	Influenza B	5	430	NA ^b^	NA ^b^
	Vaccinees	1 (20.0)	126 (29.3)		
	Unvaccinees	4 (80.0)	304 (70.7)		
	Overall influenza	25	1105	-144.8 (-444.5 to -10.1)	-17.2 (-218.9 to 56.9)
	Vaccinees	14 (56.0)	378 (34.2)		
	Unvaccinees	11 (44.0)	727 (65.8)		

CI, confidence interval; ICU, intensive care unit; NA, not assessed

^a^ Influenza-related complications include pneumonia, asthma exacerbation, chronic obstructive pulmonary disease exacerbation, myocardiac infarction, acute renal failure, encephalopathy, and rhabdomyolysis.

^b^ The vaccine effectiveness for preventing hospitalization to ICU in influenza B subgroup was not assessed because the influenza B patients hospitalized to ICU was too small (n = 5).

## Discussion

The study findings indicated that the 2011–2012 seasonal influenza vaccine was not effective in preventing laboratory-confirmed influenza, especially for influenza A. In addition, the vaccine did not demonstrate a statistically significant effectiveness for preventing influenza-related complications, including pneumonia, hospitalization or death. This finding was different from a previous study performed during the 2010–2011 season [8]. The main circulating influenza strain in the 2010–2011 season was the A/H1N1 2009pdm virus. During the 2011–2012 influenza season, however, 51.5% of the circulating influenza strains in Korea were influenza A, and most of them were H3N2 according to a report of the Korea Centers for Disease Control and Prevention (KCDC) [10]. Hospital-based Influenza Morbidity and Mortality (HIMM) surveillance found the prevalence to be 71.6% influenza A/H3N2, 0.2% influenza A/H1N1, 8.2% unspecified influenza A, and 20.0% influenza B during that year [11]. These findings indicated that the suboptimal VE shown in this study reflected VE for preventing laboratory-confirmed influenza with A/H3N2 viruses.

The reason for suboptimal VE may be explained by a poor match between the vaccine strain and the circulating A/H3N2 viruses. The information on the match between vaccine strain and circulating viruses during the 2011–2012 influenza season in Korea is lacking, but the World Health Organization reported that the circulating A/H3N2 viruses in the 2011–2012 season were antigenically heterogenous [12]. Moreover, most of the circulating influenza A/H3N2 viruses were antigenically and genetically indistinguishable from the vaccine virus A/Perth/16/2009 (H3N2) and were more closely related to A/Victoria/361/2011(H3N2)-like reference viruses. Current vaccines containing A/Perth/16/2009(H3N2) antigens stimulated antibodies that were lower than the most recent influenza A/H3N2 viruses.

Almost all of the influenza vaccines used in Korea are the traditional egg-based, unadjuvanted inactivated vaccines. Limited effectiveness of the traditional influenza vaccine has been well documented, especially in the elderly [13–15]. Adjuvanted influenza vaccine is known to have a cross-protective effect. Considering the difficulty in predicting the circulating influenza viruses each season and the limitation of traditional unadjuvanted inactivated vaccines, broader use of adjuvanted influenza vaccines should be considered in Korea. In addition, it has been reported that the egg-based influenza vaccine could have suboptimal effectiveness during the adaptation of the vaccine strain to eggs [16]. Therefore, the cell-based influenza vaccine could be another, better option. However, this needs to be proven using a proper field trial.

Some previous studies have evaluated the effectiveness of the 2011–2012 influenza vaccine. The I-MOVE study performed in Europe showed a statistically insignificant VE among all ages [17]. The study performed with the pooling of the data obtained through a European network of hospitals showed a low VE against hospitalized confirmed influenza [18]. The study performed with the US Influenza Vaccine Effectiveness Network showed a modest overall VE and low effectiveness against the predominant A (H3N2) virus [19]. In contrast, the study performed in the US showed high and statistically significant VE [20]. The study performed in Canada also showed substantial VE [21]. The diversity of the results may be due to the different study method, the diverse match between circulating viruses and the unique vaccine strains found in each region.

This study had several limitations. First, some of the control participants may have been influenza patients, although every effort was made to minimize this limitation. In this study, the control participants used to determine the VE for preventing laboratory-confirmed influenza were age group-matched patients who visited the same hospital with ILI within 48 hours of symptom onset but for whom laboratory tests were negative for influenza. A limitation in using these control participants was that some of the participants were true influenza patients with false-negative results, especially with the RAT [22–25]. This limitation could have produced a biased estimation of the effectiveness of the influenza vaccine. The RAT is known to have low sensitivity, especially for patients who take the test after ≥3 days of symptom onset [22]. Therefore, to minimize the false negative results, control participants were restricted to patients who visited the same hospital with ILI within 48 hours of symptom onset. This type of control was proven to be appropriate in the previous study [8]. This method of control selection might cause another type of bias. That is, the healthcare seeking behavior related to vaccination status can affect the result by restricting the control participants to patients who visited the same hospital with ILI within 48 hours of symptom onset. Nevertheless, the method of control selection was thought to be more desirable in the view of minimizing the false negative. It is also be a limitation that a significant number of patients were excluded due to an obscure influenza vaccination history. Second, this analysis was performed on symptomatic patients who visited university hospitals. Therefore, the characteristics of patients utilizing university hospitals could have affected the results of the study. In addition, all four hospitals participating in the study is located in the metropolitan area. It means that the four hospitals cannot be representative of Korea. Third, the influenza vaccination history was checked by reviewing the medical records and interviews with the patients. However, there may have been a recall bias in the interviews with the patients. Lastly, it would be appropriate to apply the results of this study to the traditional unadjuvanted influenza vaccine. Other types of influenza vaccines, such as a live vaccine or an adjuvanted vaccine, may show different results.

In conclusion, the 2011–2012 seasonal influenza vaccine was not effective for preventing laboratory-confirmed influenza or influenza-related complications in adult Korean populations. The use of next-generation influenza vaccines should be considered to improve the effectiveness of the influenza vaccine.
